# Case Report: Metagenomic Next-Generation Sequencing in Diagnosis of Disseminated Tuberculosis of an Immunocompetent Patient

**DOI:** 10.3389/fmed.2021.687984

**Published:** 2021-07-12

**Authors:** Yuanting Ye, Naibin Yang, Jingying Zhou, Guoqing Qian, Jinguo Chu

**Affiliations:** ^1^School of Medicine, Ningbo University, Ningbo, China; ^2^Department of General Practice, Ningbo First Hospital, Ningbo, China; ^3^Department of Infectious Disease, Ningbo First Hospital, Ningbo, China; ^4^Department of Hematology, The Second Affiliated Hospital and Yuying Children's Hospital of Wenzhou Medical University, Wenzhou, China

**Keywords:** disseminated tuberculosis, metagenomic next-generation sequencing, immunocompetence, extrapulmonary tuberculosis, diagnosis

## Abstract

Disseminated tuberculosis (TB) is a rare disease and mainly occurs in immunodeficient patients. It is marked by hematogenous or lymphatic dissemination of *Mycobacterium tuberculosis*, causing tuberculous infection involving any organ system. Here, we report a case of disseminated TB involving lung, liver, spine, mediastinum, and prostate in an immunocompetent man. The present patient found a hepatic mass without any symptom during health examination. In the next 2 years, further examinations revealed multiple lesions in the lung, mediastinum, spine, and prostate. Imaging examinations, such as contrast-enhanced abdominal CT, F-18 FDG-PET/CT, and radionuclide bone scan, suggested the diagnosis of malignancy or metastatic tumor. Furthermore, histopathological results of the biopsies of the hepatic mass, mediastinal mass, and prostatic mass demonstrated granulomatous inflammation. Therefore, metagenomic next-generation sequencing (mNGS) was utilized to confirm the diagnosis. *Mycobacterium tuberculosis* complex was simultaneously detected in the spinal surgical resection specimens and bronchoalveolar lavage fluid (BALF), indicating the diagnosis of disseminated TB. mNGS is an emerging molecular diagnostic technology, and its application in disseminated TB has been rarely reported. We highlight that disseminated TB should be considered even in an immunocompetent patient, and mNGS can be performed when the diagnosis is difficult.

## Introduction

Tuberculosis (TB) is a widespread infectious disease caused by *Mycobacterium tuberculosis* (MTB), which can involve any organ system and mostly the lungs (1, 2). Extrapulmonary infection of MTB was also reported in recent decades, including lymph glands, pleura, bones, joints, urogenital tract, and central nervous system (CNS) (3–7). Besides, disseminated TB is a rare form accounting for about 1–5% of all TB cases and defined as tuberculous infection involving two or more non-adjacent body sites via hematogenous or lymphatic spread of MTB from the primary lesion (8). The dissemination mainly occurs in patients with risk factors including HIV immunodeficiency, long-term use of immunosuppressants, poorly controlled diabetes, hematologic diseases, and alcohol abuse (9). However, it is less likely to appear in immunocompetent individuals.

Metagenomic next-generation sequencing (mNGS) is a new molecular diagnostic technology. mNGS application in TB of single organ has been reported, such as pulmonary TB, osteoarticular TB, and tuberculous meningitis, but rarely in disseminated TB until now (10–12). Here, we report a case of disseminated TB with a hepatic mass as the first manifestation. The patient failed to be diagnosed despite several biopsies of liver mass, mediastinal mass, and prostatic nodules. With the application of mNGS, the patient was finally diagnosed with disseminated TB.

## Case Presentation

**Figure 1:**
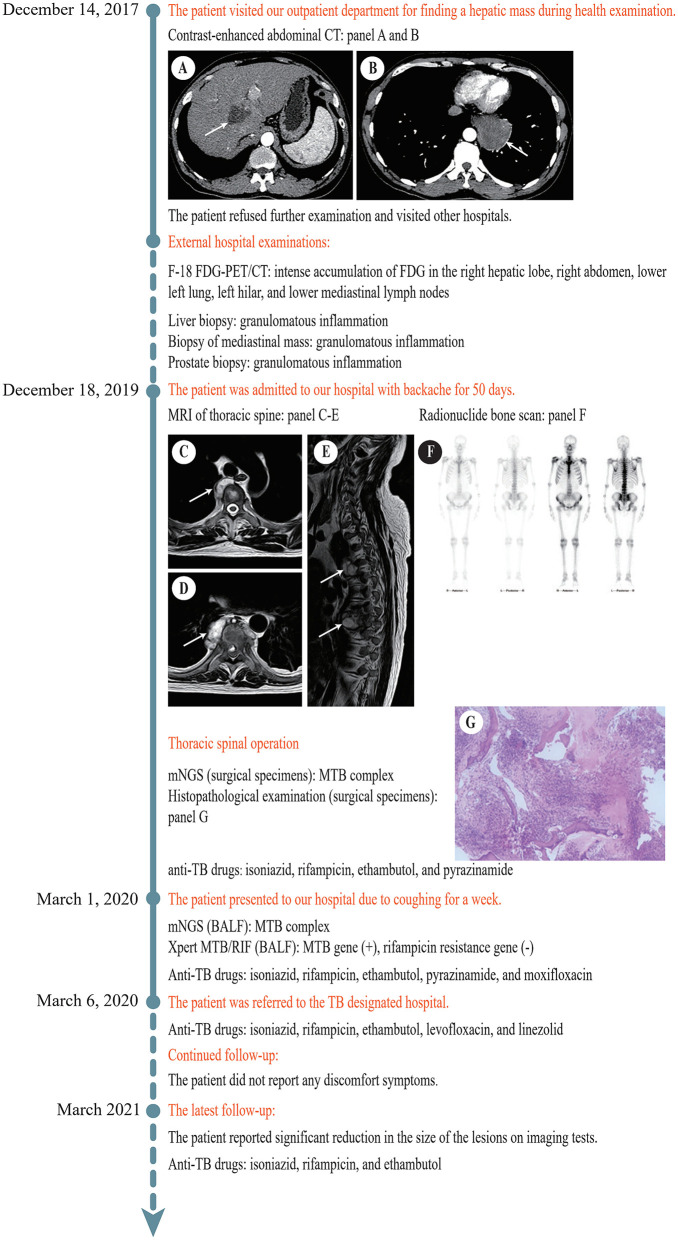
Timeline of the diagnosis and treatment process. **(A)** Contrast-enhanced abdominal CT revealed a hypodense lesion (white arrow) with mild to moderate enhancement in the right hepatic lobe near hepatic hilar. **(B)** Contrast-enhanced abdominal CT revealed a soft tissue density lesion (white arrow) with heterogeneous enhancement in the left posterior mediastinum. **(C)** MRI of thoracic spine revealed a soft tissue lesion (white arrow) at T3/T4. **(D)** MRI of thoracic spine showed a soft tissue lesion (white arrow) at T7/T8. **(E)** MRI of thoracic spine displayed multiple bone destructions (white arrows) at T3/T4 and T7/T8 with surrounding soft tissue lesions. **(F)** Radionuclide bone scan showed that the metabolism of vertebral bodies of T4 and T8 was abnormal. **(G)** Histopathological examination of the lesion at T7/T8 showed caseous necrosis with a significant number of inflammatory cells and multinucleate giant cells. CT, computed tomography; F-18 FDG-PET, 18F-fluorodeoxyglucose positron emission tomography; MRI, magnetic resonance imaging; T, thoracic vertebra; BALF, bronchoalveolar lavage fluid.

On December 14, 2017, a 51-year-old man visited our General Internal Medicine department for finding a hepatic mass without any discomfort during health examination. The entire process of diagnosis and treatment is briefly depicted in Figure 1. The patient claimed no history of hepatitis or TB and no family history of liver cancer. Physical examination was unremarkable. Detailed laboratory examination data are presented in Table 1. Contrast-enhanced abdominal computed tomography (CT) showed a hypodense lesion with mild to moderate enhancement in the right hepatic lobe near hepatic hilar, which indicated the possibility of cholangiocellular carcinoma; in addition, a soft tissue density lesion with heterogeneous enhancement in the left posterior mediastinum suggested the possibility of neurogenic tumor (Figures 1A,B).

**Table 1:** Laboratory examination results of the patient.

**Items**	**Values**	**Reference range**
White blood cell count (×10^9^/L)	6.19	3.50–9.50
Neutrophil count (×10^9^/L)	3.9	1.8–6.3
Lymphocyte count (×10^9^/L)	1.2	1.1–3.2
Monocyte count (×10^9^/L)	0.9	0.1–0.6
Red blood cell count (×10^12^/L)	4.02	4.3–5.8
Hemoglobin (g/L)	129.0	130.0–175.0
Platelet count (×10^9^/L)	273.0	125.0–350.0
Erythrocyte sedimentation rate (mm/h)	10	<21
Alanine aminotransferase (U/L)	33	9–50
Aspartate aminotransferase (U/L)	27	15–40
γ-Glutamyl transpeptidase (U/L)	252	10–60
Alkaline phosphatase (U/L)	183	45–125
Total bilirubin (μmol/L)	12.9	3.4–20.5
Direct bilirubin (μmol/L)	3.8	0.0–6.84
Indirect bilirubin (μmol/L)	9.1	1.7–13.7
Carcinoembryonic antigen (ng/ml)	1.48	0.00–5.00
Alpha-fetoprotein (ng/ml)	3.11	0.00–9.00
Hepatitis B surface antigen (IU/ml)	0	0.00–0.05
Hepatitis B surface antibody (mIU/ml)	18.16	0.00–10.00
Hepatitis C virus antibody	Negative	Negative
Human immunodeficiency virus antibody	Negative	Negative
Treponema pallidum antibody	Negative	Negative

In the following 2 years, he had visited several hospitals and had underwent a huge number of blood tests, CT scans, and magnetic resonance imaging (MRI) examinations. Mantoux test was positive (24 ×22 mm). The results of T-SPOT.TB tests elevated progressively from 14 to 84 pg/ml. Repeated sputum smear examinations for acid-fast bacilli (AFB) were all negative. Chest CT displayed multiple small lung nodules and a left posterior mediastinal mass. 18F-fluorodeoxyglucose positron emission tomography (F-18 FDG-PET)/CT showed intense accumulation of FDG in the right hepatic lobe, right abdomen, lower left lung, left hilar, and lower mediastinal lymph nodes, suggesting the possibility of malignancy. MRI showed multiple cystic nodules in the prostate. Histopathological examinations of specimens obtained by fine-needle aspiration biopsy from the liver mass, mediastinal mass, and prostatic nodules suggested granulomatous inflammation, and special stains including acid-fast staining, Grocott's methenamine silver (GMS), Periodic acid-Schiff (PAS), and gram were all negative. The diagnosis remained unclear.

On December 18, 2019, the patient was admitted to our hospital again with backache for 50 days. MRI of thoracic spine demonstrated multiple bone destructions at T3/T4 and T7/T8 with surrounding soft tissue lesions, suggesting spinal TB with cold abscesses (Figures 1C–E). Radionuclide bone scan demonstrated that the metabolism of vertebral bodies of T4 and T8 was abnormal, and metastatic bone tumors were considered (Figure 1F). He underwent a thoracic spinal operation. Considering the possibility of TB, the surgical specimens were subjected to both mNGS and histopathological examination. The process of mNGS is provided in the Supplementary Material. The mNGS revealed 25 total reads of MTB complex (Table 2). Significantly, because of the high internal homology and relatively insufficient coverage, the species within the MTB complex was not determined (13). Meanwhile, histopathological examination showed caseous necrosis with a significant number of inflammatory cells and multinucleate giant cells (Figure 1G). Therefore, the patient was diagnosed with spinal TB and immediately treated with anti-TB drugs including isoniazid, rifampicin, ethambutol, and pyrazinamide.

**Table 2:** Result of mNGS of the spinal surgical resection specimens.

**Genus**			**Species**	
**Name**	**Sequence number**	**Relative abundance (%)**	**Name**	**Sequence number**
*Mycobacterium tuberculosis complex*	25	1.0	**/**	**/**
*Staphylococcus*	38	3.9	*S. haemolyticus*	23
*Moraxella*	13	1.2	*M. osloensis*	13
*Propionibacterium*	7	0.5	*P. acnes*	3

On March 1, 2020, the patient presented to our hospital for the third time due to coughing for a week. His bronchoalveolar lavage fluid (BALF) was obtained by fiberoptic bronchoscopy for mNGS and Xpert MTB/RIF assay. The process of Xpert MTB/RIF is provided in the Supplementary Material. MTB complex was successfully detected by mNGS. In addition, the result of Xpert MTB/RIF was positive for MTB gene and negative for rifampicin resistance. Based on the available evidence, it was eventually considered that the patient suffered from disseminated TB with systemic multi-organ involvement, including the lung, spine, mediastinum, liver, and prostate. To enhance the treatment effect, moxifloxacin was added to the original anti-TB treatment regimen.

According to the national laws and regulations, the patient was subsequently referred to the TB designated hospital for systematic treatment because of intrapulmonary TB. Through telephone follow-up, we learned that pyrazinamide was discontinued because of hyperuricemia shortly after the referral, and then the patient received anti-TB treatment with isoniazid, rifampicin, ethambutol, levofloxacin, and linezolid for 1 year. Diammonium glycyrrhizinate, which protects the liver, was continuous in the course of anti-TB therapy. The anti-TB drug regimen was well-tolerated and achieved a remarkable effect. At the latest follow-up, the patient reported significant reduction in the size of the lesions on imaging tests, reconfirming the diagnosis of TB, and started receiving anti-TB treatment with isoniazid, rifampicin, and ethambutol. He thanked us for our contribution to the diagnosis.

## Discussion

The clinical presentation of disseminated TB can vary greatly according to the involved organ system. It can present with atypical extrapulmonary symptoms as the first manifestation, often leading to a diagnostic dilemma. In our case, the patient was immunocompetent and came to our hospital due to a mass in the liver without any typical symptoms. For most clinicians, malignancy and infectious diseases are primary considerations among the broad differential diagnosis. Their identification needs evidence from additional tests.

WHO reported that nearly 30% TB cases failed to be diagnosed (14). The hepatic TB are largely non-specific and exhibit an extensive overlap with more common primary or metastatic liver carcinoma under image findings (15). Initially, this case was misdiagnosed as hepatic carcinoma according to the contrast-enhanced abdominal CT scan. Recently, F-18 FDG-PET/CT has emerged as an effective tool for the diagnosis and evaluation of malignant tumors. However, it is difficult for F-18 FDG-PET/CT to distinguish between inflammatory and malignant lesions because of the strong accumulation of F-18 FDG in both tissues (16). In our case, the F-18 FDG-PET/CT report was more favorable of malignancy vs. inflammation. Regrettably, due to the influence of the above imaging results or the lack of attention by clinicians to infectious diseases, the specimens from three biopsies were just collected for histopathological examinations, but no pathogenic detections, which led to diagnostic delay and worse clinical course.

At present, the dominating methods of diagnosing disseminated TB are still pathogen culture, AFB smear, nucleic acid amplification test (NAAT), and histopathological examination (17). MTB culture is currently considered as the gold standard of diagnosis, but low sensitivity and long culture time make it cannot meet the need of rapid clinical diagnosis (18). AFB smear, as a simple and fast tool, is unsatisfying due to lower sensitivity and specificity (19, 20). Just like the biopsies in our case, histopathological examination sometimes only shows granulomas without typical caseous necrosis, which is non-specific for the diagnosis of TB. PCR is a valuable diagnostic technique, but it always requires presupposing specific pathogens, which sometimes cannot detect rare pathogens and mixed infections (21). Additionally, Xpert MTB/RIF, a new automated molecular test, has been endorsed by WHO for the initial detection of TB and rifampicin resistance (14). Nevertheless, the sensitivity of Xpert MTB/RIF varies greatly with the type of extrapulmonary samples (22, 23). Significantly, mNGS exhibits better diagnostic performance than Xpert MTB/RIF in detecting MTB among various samples. Mutual combination can stimulate further elevation of the performance (13, 24).

mNGS, an unbiased culture-independent high-throughput sequencing technology, has shown a considerable clinical application prospect in diagnosis of infectious diseases over the past few years. In our case, mNGS finally unmasked TB after 2 years of delayed diagnosis and misdiagnosis. Notably, several prospective studies have indicated that mNGS shows an excellent diagnostic performance in suspected TB patients with 62–87.5% sensitivity for intrapulmonary samples, 47.4–60% sensitivity for extrapulmonary samples, and almost 100% specificity in various samples (13, 24, 25). In contrast to some traditional microbiological tests designed to specifically detect just one or a limited spectrum of known pathogens at a time, mNGS can simultaneously detect thousands of pathogens, including bacteria, fungi, viruses, and even parasites, without requiring any prior knowledge (26). Additionally, the turnaround time of mNGS, usually 2–3 days, is shorter than several weeks of traditional MTB culture, which might benefit clinicians in making rapid diagnosis and guiding precise antimicrobial therapy. On the other hand, mNGS can obtain all nucleotide sequence information in clinical samples, including nucleotide sequences from human, contaminant during operations, harmless parasitic microbes, and true pathogens, thus can create challenges in data processing and interpretation of results (27). MTB, as one of the intracellular bacteria, is characterized by releasing fewer nucleic acids into extracellular environment, resulting in a small number of sequences detected, which may also lead to the difficulty of detecting MTB by mNGS (28). The detection of resistance genes and the strain discrimination of the MTB complex are hard to achieve clinically because they highly depend on genome coverage, and combining with targeted PCR might be a good choice (13). The combination of mNGS and Xpert MTB/RIF shows great potential in detecting MTB, which is superior to traditional pathogenic detection methods. In our case, MTB was reconfirmed by fiberoptic bronchoscopy combining with mNGS and Xpert MTB/RIF, meeting the requirement for etiology diagnosis and proving the clinical value of the combined application. Thus, mNGS alone or in combination with Xpert MTB/RIF provides clinicians with a reliable TB diagnosis method.

In conclusion, we emphasize that disseminated TB should also be considered in an immunocompetent patient even when imaging results favor malignant lesions based on the experience from this case. It is essential for clinicians to perform pathogenic examinations in time when suspicious specimens are encountered, and mNGS can be used as a valuable complementary means for TB diagnosis.

## Data Availability Statement

The original contributions presented in the study are included in the article/Supplementary Material, further inquiries can be directed to the corresponding author/s.

## Ethics Statement

Ethical review and approval was not required for the study on human participants in accordance with the local legislation and institutional requirements. The patient provided his written informed consent to participate in this study.

## Author Contributions

YY and NY analyzed and interpreted the clinical data. YY, NY, and JZ drafted the manuscript. GQ and JC revised the manuscript. All authors have read and approved the final manuscript.

## Conflict of Interest

The authors declare that the research was conducted in the absence of any commercial or financial relationships that could be construed as a potential conflict of interest.
