# Supplementary material for: Case Report: Metagenomic Next-Generation Sequencing in Diagnosis of Disseminated Tuberculosis of an Immunocompetent Patient
Source: Front Med (Lausanne). 2021 Jul 12;8:687984. doi: 10.3389/fmed.2021.687984 (PMC8310911; doi:10.3389/fmed.2021.687984)
Supplement: Supplementary file 1 [file Data_Sheet_1.docx]

**Supplementary Materials**

Metagenomic Next-Generation Sequencing

Nucleic acids were extracted using the QIAamp DNA Mini Kit (Qiagen, Hilden, Germany). Libraries were constructed using the NEBNext Ultra DNA Library Prep Kit (NEB, Ipswich, MA, USA). Sequencing was performed on a HiSeq 2500 Sequencer (Illumina, San Diego, CA). After filtering out short, low-complexity, and low-quality reads, the clean reads were mapped to the human reference genome (hg38) using bowtie2, which removed human host contamination. The remaining sequencing data were aligned to the NCBI non-redundant nucleotide database by using BLASTn. After alignment, the parameters of different microorganisms were classified and recorded according to bacteria, fungi, viruses, and parasites. Then, the final report was obtained.

Xpert MTB/RIF assay

Bronchoalveolar lavage fluid (BALF) was collected from the patient. 4ml of BALF was placed in a leak-proof container. Equal volume of processing solution was added and mixed thoroughly. 2 mL of the mixture was obtained with a new pipette and added to the Xpert cartridge. The cartridge was placed into the detection module of GeneXpert GX-XVI to start automatic detection. About two hours later, we got the test result.
